# Functional assessment of Phospholipase D1 using wild‐type, transgenic AD mice, and human spheroid models: A spatial and temporal mechanism for the progression of neurodegeneration

**DOI:** 10.1002/alz.089269

**Published:** 2025-01-03

**Authors:** Sravan Gopalkrishna Shetty Sreenivasa Murthy, Chandramouli Natarajan, Klarissa H Garza, Shaneilahi M Budhwani, Sarah Bosworth, Joshua Currie, Sanjana Mohanty, Malav S Mallipudi, Kyle S Sheth, Evan Wong, Poonam Shah, Thomas A Green, Marlene E Villarreal Juarez, Agenor Limon, Emily Lee, Jiajing Zhang, Marc Ferrer, Balaji Krishnan

**Affiliations:** ^1^ University of Texas Medical Branch, Galveston, TX USA; ^2^ National Center for Advancing Translational Sciences, National Institute of Health, Bethesda, MD USA

## Abstract

**Background:**

Alzheimer’s disease (AD) is the memory‐related neurodegenerative disorder, contributing to 70% of the cases globally. Synaptic dysfunction is a well‐known early event that causes progressive cognitive decline in AD. The latest AD therapeutics on the forefront only offer a moderate symptomatic relief with significant off‐target effects. Therefore, understanding the mechanism for AD pathogenesis and developing novel therapeutic targets are urgently needed. Our lab has recently reported an anomalistic increase in phospholipase D isoform 1 (PLD1), that breakdown phospholipids in AD postmortem brain samples, compared to control subjects. Moreover, the effect of elevated PLD1 driven by amyloid‐β and tau deposits has been well‐established in wild type and in 6‐month‐old 3xTg‐AD model mice. In the present study, we assess the novel role of PLD1 in modulating cellular mechanisms involved in synaptic dysfunction in AD.

**Method:**

Here, we studied the spatial and temporal expression of PLD1 in 3xTg‐AD model mice, treated with a small molecule PLD1 inhibitor (VU0155069), in an age‐dependent manner. Furthermore, the brain‐region specific mechanisms of PLD1 were evaluated by utilizing adeno‐associated viral 2 (AAV2) vectors via intracerebroventricular route in 18‐ and 24‐month‐old wild‐type and 3xTg‐AD model mice. Following VU0155069/AAV2 administration, the mice cohorts were subjected to behavioral studies specific to learning and memory, such as Y‐maze, novel object recognition (NOR), and elevated plus maze. Synaptic dysfunctions were studied using high frequency stimulation long‐term potentiation (HFS‐LTP), by conventional electrophysiology and multi‐electrode array (MEA). Finally, the synaptic strength in frozen synaptosomal (P2) fractions was determined by previously standardized novel *in vitro* assay called the Fluorescence‐Assisted Single Synaptosome‐Long Term Potentiation (FASS‐LTP). Morphological changes in the synapse were assessed using ImageJ and IMARIS following Golgi‐Cox staining, a gold standard for measuring dendritic spine integrity. To deduce the underlying mechanisms, we used brain spheroids developed from human tissue to test the effects of PLD1 inhibition.

**Result:**

In WT aged mice we noted differential effects of PLD1 over expression and attenuation. Additionally, we corroborate our results with diseased aging seen in 3xTg‐AD using pharmacological and molecular approaches with AAV2 vectors.

**Conclusion:**

Our research provides a novel insight into how PLD1 contributes to progressive functional deficits associated with synaptic dysfunction by impinging on critical cellular signaling events compromised in early and late stages of Alzheimer’s disease.

